# The Effect of a Multi-Level Intervention on the Initiation of Antiretroviral Therapy (ART) among HIV-Infected Men Who Inject Drugs and Were Diagnosed Late in Thai Nguyen, Vietnam

**DOI:** 10.1371/journal.pone.0161718

**Published:** 2016-08-31

**Authors:** Carla E. Zelaya, Nguyen Le Minh, Bryan Lau, Carl A. Latkin, Tran Viet Ha, Vu Minh Quan, Thi Tran Mo, Teerada Sripaipan, Wendy W. Davis, David D. Celentano, Constantine Frangakis, Vivian F. Go

**Affiliations:** 1 Johns Hopkins Bloomberg School of Public Health, Department of Epidemiology, 615 N. Wolfe Street, Baltimore, Maryland 21205, United States of America; 2 Centre for Preventive Medicine of Thai Nguyen, 971 Duong Tu Minh Road, Thai Nguyen City, Vietnam; 3 Johns Hopkins Bloomberg School of Public Health, Department of Health, Behavior, and Society, 624 N. Broadway, Hampton House 737, Baltimore, Maryland 21205, United States of America; 4 University of North Carolina, No 6, Lane 76, Linh Lang Street, Hanoi, Vietnam; 5 University of North Carolina at Chapel Hill Gillings School of Global Public Health, Department of Health Behavior, 361 Rosenau Hall, Campus Box 7440, Chapel Hill, NC 27599, United States of America; 6 Johns Hopkins Bloomberg School of Public Health, Department of Biostatistics, 615 N. Wolfe Street, Room E3642, Baltimore, Maryland 21205, United States of America; University of Washington, UNITED STATES

## Abstract

**Background:**

In Vietnam, an estimated 256,000 people are living with HIV, and 58% of HIV-infections reported are among people who inject drugs (PWID). While antiretroviral therapy (ART) is widely available in Vietnam, marginalized hard-to-reach male PWID, demonstrate significantly reduced and delayed access to ART.

**Methods:**

We investigated the effect of a randomized four-arm multi-level intervention trial on ART initiation among male PWID. Our analysis was conducted among a subset of trial participants (n = 136), who were newly diagnosed as HIV-infected, treatment naïve, and eligible for ART (baseline late diagnosis). The trial arms included: 1, standard of care (HIV testing and counseling); 2, structural-level intervention (door-to-door communications and community video screenings); 3, individual-level intervention (counseling plus group support); and 4, individual-level plus structural-level intervention. In a time-to-event analysis, we used a non-parametric approach for competing risks to estimate cumulative incidence function (CIF) for ART initiation (event of interest) by arm and the difference in CIF for each trial arm as compared to Arm 1. Follow-up was conducted at 6, 12, 18 and 24 months. Data collection occurred from 2009 to 2013.

**Findings:**

By 24-months, 61.0% initiated ART, and 30.9% had died prior to ART initiation. In the first 6 months, participants in arm 4 (individual plus community intervention) had a 28% (95% confidence interval (CI): 6–50%) increased probability of initiating ART. Despite increasing coverage of ART in all arms throughout follow-up, participants in arm 4 retained a 31% (95% CI: 5–56%) increased probability of initiating ART. The individual and community components of the intervention were only effective when delivered together.

**Conclusions:**

Marginalized, hard-to-reach men, who do not routinely engage in HIV services, and therefore come into care late, may benefit significantly from both individual counseling and group support, in combination with community-focused stigma reduction, when being referred and attempting to initiate urgently needed ART.

## Introduction

Globally, it is estimated that there are approximately 12.7 million people who inject drugs (PWID) and approximately 1.7 million of these are HIV-infected.[1] Injection drug use is the primary driver of HIV epidemics in areas of Eastern Europe, Central and Southeast Asia. Globally, Vietnam is thought to have the sixth largest population of people who inject drugs (PWID), with 290,000 people estimated to be injecting drugs in 2009.[2] As of 2014, there are an estimated 256,000 people living with HIV in Vietnam[3, 4], with PWID accounting for 58% of infections.[5]

PWID are known to have poorer outcomes when HIV-infected than others,[6] in part due to diminished and delayed access to ART,[6–13] and therefore need programs that are tailored for their unique needs. The three interventions deemed effective in reducing transmission of and treating HIV among PWID are: opioid substitution therapy (OST), antiretroviral therapy (ART) and needle and syringe programs (NSP).[2]

In Vietnam, the availability of effective services has encouragingly increased in recent years, with the introduction of NSP in some provinces,[2, 14] and the expansion of Methadone Maintenance Therapy (MMT) after a successful pilot started in 2008.[15] The government has committed to national ART expansion with 37% of people living with HIV receiving ART nationally in 2014,[2, 16] and has named PWID a priority group in the national plan.[17] National estimates of ART coverage among PWID are unavailable,[2, 5] however one prior study in Vietnam observed that PWID were 2.13 times more likely to initiate ART very late (CD4 count ≤100 cells/mm³) and this late initiation was significantly associated with death.[18]

Recent evidence has demonstrated the importance of tailoring and rolling out combination prevention strategies for PWID and other marginalized groups, such as integrating ART and OST services, and paying particular attention to removing the barriers that these groups face in accessing and utilizing effective services.[8, 19–21] Expanding ART coverage for PWID is increasingly an issue of access rather than availability in many settings such as Vietnam.

Although in many countries injection drug use is stigmatized, in Vietnam, the government programs to prevent ‘social evils’, such as injection drug use, were purposely linked to HIV control programs.[22, 23] This fear based approach, likely exacerbated stigma already attached to injection drug use with HIV-related stigma.[22–24] Pervasive internalized, anticipated, and experienced stigma related to injection drug use and HIV has proved a major barrier to PWID initiating ART in Vietnam and other similar settings globally.[21, 25–27] For example, PWID report lack of ART initiation due to fatalistic attitudes and lack of family and community support.[25] PWID in some settings including Vietnam, are required to register with authorities upon seeking treatment,[23, 26] and also perceive and fear that they will not be treated well or confidentially in health care settings.[21, 23, 25] In addition, many people living with HIV in Vietnam hold the inaccurate belief that they do not need to be treated until they feel sick.[21] Therefore many PWID in Vietnam fail to engage in the HIV care continuum until a very late stage, when symptoms are prevalent and treatment is urgent.[21]

In 2008 our team developed a multi-level prevention intervention, named ACCEPT PROJECT, to reduce HIV transmission and increase support and coping, among HIV-infected PWID, by addressing known barriers to prevention and care at the individual and community level. The main barriers addressed were a) HIV- and drug-related stigma and social isolation, which prevent disclosure and subsequent adoption of risk reduction strategies, and b) the lack of in-depth knowledge of HIV disease and effective sexual and injecting risk reduction strategies. The effectiveness of this intervention was evaluated through a randomized controlled trial among HIV-infected PWID and community members. The trial compared standard HIV voluntary counseling and testing (VCT) to an intervention that adds a continuum of psychosocial support for HIV-infected PWID, and community stigma reduction. The primary and secondary outcomes of the trial for the full cohort of HIV infected participants are reported elsewhere, and include sexual and injecting risk behaviors, disclosure, stigma and social support.[28]

Given the delayed uptake of ART and reported barriers among PWID in Vietnam and elsewhere, we investigated the effect of the described multi-level intervention on initiation of ART among a subset of trial participants who were newly diagnosed as HIV-infected, treatment naïve, and eligible for therapy at the time of baseline data collection. We conducted a competing risks analysis as the likelihood of advanced disease and dying was high in this subgroup, primarily due to clinically late diagnosis and no history of ART use. We hypothesized that both the individual-level intervention and structural-level community stigma reduction activities (separately and together) given to HIV-infected participants and their corresponding communities encouraged initiation in ART.

## Methods

### Ethics Statement

Written informed consent was obtained from all participants. The study and consent procedures were approved by the ethical review committees at the Johns Hopkins Bloomberg School of Public Health and the Thai Nguyen Center for Preventive Medicine.

### Study Site

The site of this study was the northern province of Thai Nguyen in Vietnam (S1 Fig). Thai Nguyen has a population of approximately 1,173,000, an estimated population of 5208 PWID.[29] Substance use is a significant public health problem in Thai Nguyen, consisting primarily of heroin use like many other provinces in Vietnam. According to national sentinel HIV surveillance data in 2013, HIV prevalence among PWID highest in Thai Nguyen province (34%), followed closely by Lai Chai (27.7%) and Hanoi (24%) provinces respectively, in contrast to the lowest national HIV prevalence estimate of less than 11.6% among PWID since 1997.[4]

### Trial Design and Intervention

The trial had a factorial design with four arms: Arm 1—the standard of care (HIV testing and counseling, HTC); Arm 2—a structural-level community stigma reduction intervention; Arm 3—a individual-level enhanced posttest counseling and skill-building support groups; and lastly Arm 4—the standard of care plus both individual-level and structural-level interventions. All participants received the standard one HIV post-test counseling session. The theories underpinning the intervention development and details of the interventions are described elsewhere,[28] however briefly: The individual-level intervention consisted of two additional HIV post-test counseling sessions, two skill-building peer support groups, and one optional support session with a self-selected support person e.g., family member. These activities aimed to reduce injecting and sexual risk (through knowledge and skill building), reduce internalized stigma, and increase disclosure, coping and support among HIV-infected PWID. The structural-level intervention consisted of two community-based programs: a) two video screenings (which challenge common misconceptions about HIV transmission and promote positive messages about HIV-infected individuals) with group discussions in the two months after baseline, and b) door-to-door communications by community volunteers (six sessions completed with 813 community members over first 12 months after baseline that disseminated HIV information and answered questions). Both community activities aim to reduce perceived and enacted HIV and drug related stigma. After the baseline assessment all HIV-infected individuals (irrespective or trial arm), who where eligible for treatment, were referred to treatment in a nearby hospital by the study physician as part of the assessment procedures. Most of the individual and structural-level intervention activities were completed in the first 6 months after baseline.

The study design consisted of 32 districts (selected out of the 180 sub-districts in Thai Nguyen with the largest number of PWID), which were then partitioned into 9 groups (2 groups with 2 districts each, and 7 groups with 4 districts each), so that within each group the sub-districts were similar in number of drug users and population (S1 Fig). A random half of the districts in each group were selected to receive the structural-level community stigma reduction intervention and the remaining districts were selected to receive the standard of care. The structural-level standard of care was messaging on HIV through village weekly public loudspeakers and educational pamphlets that were already being provided by community health stations. Within each district, regardless of structural-level assignment, a random half of the participants were assigned to receive enhanced posttest counseling and skill building and the other half received the individual-level standard of care.

Within the districts randomly assigned to the structural-level intervention, the invitations to video screenings were community-wide. However given that the districts are large (average population size of 10,000 people) the individuals invited to participate in the 6-sessions with a community volunteer were selected through a systematic geographic sampling scheme designed to include individuals who live near PWID, and therefore have the greatest likelihood of directly or indirectly influencing them.

### Participants

The study enrolled male PWID between July 2009 and January 2011. Participants were identified through a snowball sampling technique and recruited through a team of recruiters, who were typically former drug users. Inclusion criteria for the index participants were: 1) an HIV-infected diagnosis confirmed through two parallel rapid HIV tests (Determine: Abbott Laboratories, Abbott Park, IL; and Bioline: SD, Toronto, Canada); 2) able and willing to bring in an injecting network member for screening; 3) male (note: 97% of PWID in Thai Nguyen are male and female PWID typically have different risk factors.); 4) at least 18 years old; 5) had sex in the past 6 months; 6) injected drugs in the past 6 months; and 7) planned to live in Thai Nguyen for the next 2 years. After completion of baseline, all eligible index participants who agreed were enrolled into the trial as index participants and randomized (n = 455). We restricted the current analysis to 338 index participants who learnt of their HIV-infected status for first time after the baseline assessment, to ensure that a) participants analyzed had the same ‘time at risk’ to initiate ART b) had intervention just after learning they were HIV-infected (as the intervention may have a different effect on those that have been previously living with HIV), and c) were comparable in terms of history of prior engagement in HIV services. Subsequently recruited network partners were not included in this analysis as they were HIV-uninfected (as per study design). We then removed the following individuals from the analysis: those who had missing information on current ART use, or who reported being on ART regardless of previously stating they did not have a prior known HIV infection (n = 15); with a CD4 cell count higher than 200 cells/μl (n = 187). Prior to November 2^nd^ 2011 the Vietnamese Ministry of Health used a CD4 cell count of 200 cells/μl or less as a major criterion for eligibility to initiate ART. Therefore the study population in this analysis consisted of 136 injection drug users who had just learned of their HIV-infected status, were eligible for treatment at the time of baseline data collection, and who had been referred for ART for first time at the baseline visit.

### Assessment Procedures

At the baseline assessment, participants completed a survey self-reporting their prior HIV testing, their HIV status, and any prior use of ART. Participants then completed HIV-testing and counseling. Those found to be HIV-infected through study rapid tests, had their CD4 cell count measured, and received a physical exam by our study physician, who also asked the participant about prior use of ART. For those who were eligible for treatment, the study physician gave them a direct referral for treatment to a nearby hospital.

Follow-up occurred at 6, 12, 18 and 24 months. Participants were reimbursed 75,000 Vietnamese Dong, equivalent to $3.50 USD, at each visit and 5000 Vietnamese Dong ($0.23 USD) for each kilometer traveled. During the follow-up all index participants completed a survey, where they self-reported ART use in the prior 6 months. Additionally each index participant was asked to provide blood specimens to assess the CD4 cell count, and they received a follow-up physical examination by study physician, where the physician also reported ART use in prior six months. All participants who missed a visit were contacted. The study team was informed about deaths through the participants’ family members. We then conducted a verbal autopsy during which we established date of death. Event of death, was then recorded in first follow-up visit after death.

### Analysis

The data were in discrete time. In this time-to-event analysis the time of origin was baseline, and termed visit 0 in the analysis. Visits 1, 2, 3, 4 in analysis refer to follow-up visits at 6, 12, 18 and 24 months respectively. We included both a target event and competing event, and individuals were censored after either event. Mortality is considered a competing event given that it is impacted by ART initiation (the event of interest), but can also prevent the event of interest from occurring. ART initiation was the target event, and defined as first report of prior ART use in past 6 months by study physician. If physician report of ART use was missing, self-report prior ART use in past 6 months was used. Self-report of ART use had a positive predictive value greater than 92% in all visits. Among the six participants who missed a visit prior to the visit when they reported ART use for the first time (n = 6), ART initiation was assumed to have occurred in the 6 months prior to that visit where it was first reported. The competing event was death, and this was recorded by verbal autopsy. Individuals were censored after occurrence or target or competing event.

In time-to-event analysis, the occurrence of an event (e.g., mortality) that prevents the event of interest from happening is a competing risk. As individuals in this study have a non-negligible probability of death that occurs prior to initiating ART, then analytical methods are required to account for the competing event as standard survival methods such as the Kaplan-Meier survival curve will not appropriately estimate the cumulative incidence of ART initiation.[30–34] Therefore, we used a non-parametric approach for competing risks to estimate cumulative incidence function (CIF)[35, 36] for each arm and the difference in CIF for each trial arm as compared to Arm 1 –the standard of care for each visit. The CIF for initiation of ART may be interpreted as the probability of beginning treatment (prior to death, obviously) by time *t*. Similarly, the CIF for death is the probability of dying prior to ART initiation by some time point *t*. Therefore, the absolute difference in the CIF for initiation of ART (comparing each arm to arm 1) at a specific time (or visit), can be interpreted as a magnitude of the intervention effect. That is it is the excess of deficit in the probability of initiating ART for an intervention arm against standard of care. We calculate point wise 95% confidence intervals (CI) by estimating the variance for the differences in probabilities by summing the estimated variances of the treatment arm under the assumption that each trial arm are independent samples. In this analysis, the variance estimation did not account for the study design (i.e., grouping districts for randomization), however when accounted for in a similar analyses using the inferences were not affected.

To elucidate the mechanism by which the intervention was potentially directly influencing ART initiation or influencing mortality allowing for individuals to remain alive to experience initiation of ART, we calculated the cause-specific hazards (at each visit) and the estimated hazard ratios through a pooled log binomial regression, for both the target event (ART initiation) and the competing event (death prior to ART initiation) by trial arm. We allowed for time-varying relative hazard ratios, however this did not significantly improve model fit through a likelihood ratio test and therefore a parsimonious model assuming a proportional hazards assumption was used as a final model. By comparing significance of the ratios for each outcome (ART initiation and mortality), the cause-specific hazards and hazard ratios provide information on the “momentary event forces” at a point in time.[37] Thus these estimates provide information on the magnitude of the force towards a specific event for each trial arm (or relative to Arm 1).

## Results

### Description of study population

Of the HIV-infected participants (n = 455), only 26% reported that they were previously aware of their HIV-infection, 12.9% had previously initiated ART, however 40.8% were found to have a CD4 cell count equal or lower than 200 cells/μl. Of the newly diagnosed participants who were eligible for ART (based on CD4 count) in our analysis (n = 136), the mean CD4 cell count was 110.9 cells/μl and this did not differ by trial arm (Table 1). The mean age was 35 years, and the majority of participants were either currently married (50.7%) or single (37.5%), and reported working full time i.e., > = 30 hours per week (72.1%). To be eligible to participate in the study all participants had to have injected drugs in the past six months. All participants reported that most frequent drug injected was heroin, and approximately a quarter of participants had previously been in drug treatment centers (camps which are primarily compulsory with initial period of detoxification and followed by 1–2 years of vocational training), and 37.5% had previously been incarcerated at least once. Despite the low level of ART initiation observed in HIV-infected at baseline, approximately 30% of participants knew someone who had or was taking ART. None of the factors described, except ever being incarcerated, were significantly different by trial arm.

**Table 1:** Baseline characteristics of participants by trial arm (n = 136).

	Arm 1	Arm 2	Arm 3	Arm 4	Total
Sample size (N)	26	50	23	37	136
Mean CD4 count, cells/μl (Standard Deviation, SD)	114.3 (53.2)	98.1 (50.0)	127.6 (42.9)	115.7 (39.8)	110.9 (47.5)
Mean Age (SD)	34 (3.9)	35 (5.9)	36 (7.1)	35 (6.3)	34.9 (5.9)
Mean years of education (SD)	8 (3.2)	9 (3.3)	9 (2.7)	9 (2.7)	9 (2.6)
Marital Status (%)					
Single (%)	(46.2)	(38.0)	(21.7)	(40.5)	(37.5)
Currently married(%)	(42.3)	(46.0)	(60.9)	(56.8)	(50.7)
Widowed/Divorced/ Separated (%)	(11.5)	(16.0)	(17.4)	(2.7)	(11.8)
Proportion of participants who spent a night on the street, in a park, in an alley, or in an abandoned building in past 3 months. (%)	(11.54)	(16.00)	(13.04)	(10.81)	(13.2)
Proportion working full time, i.e., > = 30 hours per week. (%)	(69.2)	(70.0)	(73.9)	(75.7)	(72.1)
Proportion ever been in drug treatment (%)	(19.2)	(32.0)	(34.8)	(24.3)	(27.9)
Ever Incarcerated (prison or jail or detention center)(%)*	(53.9)	(46.0)	(26.1)	(21.6)	(37.5)
Proportion who knew someone who had taken or who was taking ART. (%)	(46.2)	(36.0)	(13.0)	(29.7)	(32.4)

*p = 0.02.

### Events

By the end of our study period, 83 (61.0%) of those newly diagnosed and eligible for treatment at baseline had initiated ART, and 42 participants were known to have died prior to initiation of ART (30.9%). Therefore 91.9% of participants had one of two events (either target or competing event), with only 11 participants (8.1%) being censored (i.e., completed study without an event) or loss to follow-up. Of the participants who reported ART initiation (in past 6 months) throughout follow-up, only 6 missed visits prior to reporting the event of interest (3 missed one visit; 2 missed two visits; 1 missed three visits). Therefore the potential for misclassifying time to event of interest was minimal.

### Intervention effect on ART initiation

**Fig 1:**
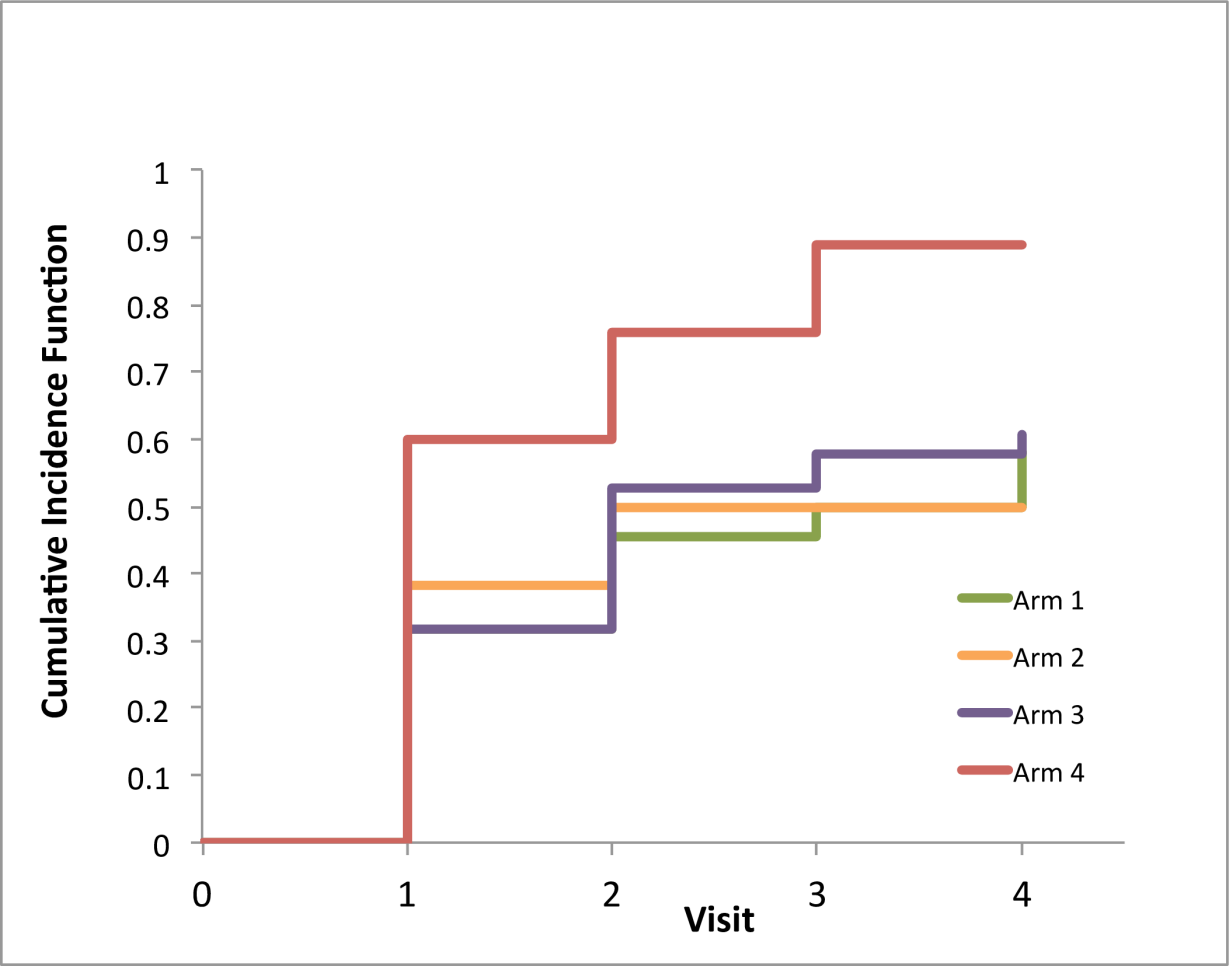
Cumulative incidence function (CIF) for ART initiation by trial arm. Arm 1—Standard of care; Arm 2- Structural-level stigma intervention; Arm 3—Individual-level intervention; Arm 4—Both structural and individual level intervention.

Fig 1 plots the cumulative incidence curves for ART initiation for each trial arm. Table 2 lists the time varying absolute difference in cumulative incidence comparing trial arms 2, 3 and 4 to arm 1, with 95% confidence intervals. These results show that in the first 6 months of follow-up participants in arm 4 had a 28% increase in probability of initiating ART compared to participants in Arm 1. In each arm, the cumulative probability of initiating ART increased every 6 months. However, the inequality between arms remained throughout the follow-up, and actually increased slightly. At 18-month, a 39% increased probability in initiating ART in arm 4 was observed. By the end of follow-up (24-months) participants in arms 1 to 3 still had a significantly lower probability of initiating ART (ranging from 0.49 to 0.60) compared to arm 4 (0.89). There was an overall 31% (confidence interval 5% to 56%) increase in the probability of ART initiation observed in Arm 4 compared to the standard of care. The cumulative incidence for ART initiation in arms 1, 2, and 3 did not statistically differ from each other throughout follow-up.

**Table 2:** Time varying difference in the non-parametric cumulative incidence function (CIF) by trial arm (arm 1 the reference). Arm 1—Standard of care; Arm 2- Structural-level stigma intervention; Arm 3—Individual-level HIV intervention; Arm 4—Both structural and individual level interventions.

Visit	1			2			3			4		
		Lo CI	Hi CI		Lo CI	Hi CI		Lo CI	Hi CI		Lo CI	Hi CI
**Arm 2**	0.06	-0.17	0.30	0.04	-0.21	0.29	0.00	-0.25	0.25	-0.08	-0.33	0.16
**Arm 3**	0.00	-0.29	0.28	0.07	-0.23	0.38	0.08	-0.23	0.38	0.02	-0.27	0.32
**Arm 4**	0.28	0.06	0.50	0.30	0.05	0.55	0.39	0.12	0.65	0.31	0.05	0.56

In order to investigate the possible mechanism of this observed intervention effect, we examined the cause specific hazards of initiating ART and death (prior to ART initiation) separately, for each visit. We also calculated the overall hazard ratios after 24 months of follow-up, using arm 1 as a reference (see Table 3). In Table 3, we can see that the cause specific hazards of death did not significantly differ by arm. However, the cause specific hazard of ART initiation was 2.27 times higher in arm 4 (combined individual and structural intervention) as compared to arm 1 (the standard of care). Taken together these data imply that individuals in arm 4 did not solely have a higher probability of initiating ART (Fig 1) by remaining alive to have the opportunity of treatment initiation.

**Table 3:** Cause specific hazard (by visit), and hazard ratio of either initiating ART (target event), or dying prior to ART initiation (competing event) by trial arm. Arm 1—Standard of care; Arm 2- Structural-level stigma intervention; Arm 3—Individual-level intervention; Arm 4—Both structural and individual level intervention.

Event	ART Initiation				Death (prior to ART initiation)			
**Cause-specific hazards by visit**	Visit				Visit			
	1	2	3	4	1	2	3	4
Arm 1	0.35	0.31	0.25	0.29	0.17	0.15	0.17	0.32
Arm 2	0.33	0.29	0.23	0.27	0.27	0.25	0.27	0.46
Arm 3	0.40	0.36	0.29	0.34	0.21	0.20	0.21	0.38
Arm 4	0.62	0.58	0.50	0.55	0.07	0.07	0.07	0.15
**Cause-specific hazard ratio, with 95% Confidence Interval (CI)**	**Estimate**	**Lo CI**	**Hi CI**		**Estimate**	**Lo CI**	**Hi CI**	
Arm 2	0.93	0.48	1.81		1.74	0.77	3.95	
Arm 3	1.19	0.56	2.55		1.31	0.49	3.50	
Arm 4	2.27	1.19	4.31		0.43	0.13	1.45	

## Discussion

Our results revealed that among HIV-infected men who inject drugs, who have not been previously diagnosed, and have a CD4 count less than 200 cells/μl and are therefore entering the HIV care continuum late in Vietnam, the first six months after receiving a positive HIV diagnosis is a critical window in time for initiating ART. Providing a comprehensive intervention which addresses HIV risk, coping, stigma and social support at the individual and structural level afforded participants in trial arm 4 a significant head start in obtaining effective medical care (28% increased probability in initiating ART in first 6 months). After this initial period, the probability of initiating ART treatment increased steadily with time (or visit) in all arms. However given the head start, participants in arm 4 remained at an advantage, with a 31% increase in the cumulative probability of initiating ART at 24-months.

None of the socio-economic factors examined, except prior incarceration, differed by trial arm, therefore we can be relatively confident that randomization balanced arms sufficiently to minimize confounding. However given that prior incarceration was significantly lower in arm 4 as compared to arm 1, future research should investigate whether stigma and discrimination associated with a history of incarceration are an additional barrier to care and probability of initiating ART. Additionally, given that the study was not powered for this sub-analysis, there could have been a difference in the probability of initiating ART over time in arms 2 and 3, as compared to arm 1, however we may not have had the sample size to observe this potentially smaller difference.

We also examined the cause specific hazards to rule out the possibility that the increase in the probability of initiating ART was purely by keeping participants alive in this sub-population with a low CD4 count. Our analysis of the cause specific hazards demonstrated that men who were in the combined intervention arm and received individual and structural-level interventions, had 2.3 times higher hazards of initiating ART over 24 months (95% confidence interval of 1.3 to 7.0), but no higher hazards of survival prior to ART initiation. Therefore, the mechanism by which some of our participants experienced a higher probability of initiating ART was likely not through increased opportunity to initiate ART (i.e., being alive), but through the effects of the interventions they were randomized to receive.

These findings are among the first evidence through randomized study designs and quantitative data to demonstrate that an intervention that addresses social and behavioral factors such as stigma at both the individual or structural level can effectively improve access to ART. Therefore these results provide an important contribution to the evidence, and warrant a careful discussion on the mechanism by which this occurred.

To understand how the individual-level enhanced counseling influenced ART initiation among our participants, we drew upon related studies conducted in PWID. A study of HIV-infected men who inject drugs in India, reported that lack of family support (e.g., being evicted from family home) and fear of societal discrimination were a major barrier to accessing treatment given that starting ART would disclose HIV status.[25] In Thai Nguyen, we previously observed that men felt despair and isolated themselves on learning they were HIV-infected due to concerns about the additional burden and negative consequences (secondary stigma) for their family, on top of that already experienced as a result of drug use.[38] However men who did disclose their status, often found it brought the family closer, as the family rallied to provide care and support.[38] This and other prior work,[39] suggests that the stigma of drug use is more pervasive than HIV-related stigma in this community. Due to these prior findings, our PWID individual counseling sessions focused on encouraging and assisting disclosure and coping in part through addressing the anticipated primary and secondary stigma and internalized stigma that men experience. Therefore our counseling (which included family and peer support sessions) may have increased self-esteem, self-efficacy, disclosure, and immediate family and peer support thereby reducing depression, and increasing the motivation and ability of an individual to seek treatment.

Others have emphasized the importance of generally mobilizing communities and increasing community awareness to reduce stigma and improve utilization of services.[40, 41] These types of interventions may increase discussions of HIV, substance use, and treatment and lead to changes in social norms. In our study, the community activities (door-to-door communications and video screenings) were targeted towards randomly selected neighbors of HIV-infected PWID, who did not themselves need the services. The success of the community activities in mobilizing a community to tackle HIV stigma in this setting is not unexpected, given the success of prior network and community approaches to family planning in Vietnam and other settings.[42] Door-to-door strategies were conducted by trained members of the community, thereby increasing the legitimacy of the messaging and community buy-in. It is also possible that the community-focused intervention reduced the PWID stigma for the families of PWIDs, and hence the family was more encouraging for the PWID to seek HIV treatment.

This body of work, and our new findings, are especially relevant to PWID who do not routinely engage in HIV prevention and care services, and are therefore diagnosed later. More research is needed to investigate how to expedite HIV testing, and peer referral and stigma reduction strategies as used in our trial may be beneficial to overcome barriers to testing. However once tested, this hard-to-reach population also requires assistance beyond a simple referral to ART treatment. Linkage to care should be supported through a combination of individual and structural approaches to behavior change.

Our results importantly demonstrate that when the intervention components were given in isolation (either individual-level enhanced counseling or structural-level community stigma reduction program) there was no observed effect on ART initiation. Without community support and a change in social norms regarding substance use and HIV, already marginalized and hard-to-reach men in need of treatment, who received counseling (including family and peer support sessions), still had the barrier of community-level stigma. This finding is not surprising in the Vietnamese setting where the influence of community at the commune level is strong (e.g., Women’s Union founded in 1930). The importance of social norms in influencing behavior has been previously described.[43] Specifically to PWID, close personal relationships among social networks have previously been successfully targeted to reduce risk behaviors by changing social norms.[44] Our results suggest that similar success can be achieved in changing health-seeking behaviors by targeting close-knit communities. It may be that men received direct encouragement to seek treatment from their neighbors newly informed on issues related to living with HIV, and/or felt more accepted and supported by their immediate community and therefore more motivated to seek treatment themselves. Either way the importance of reducing community stigma and changing social norms, in conjunction with helping an individual to cope with a new HIV diagnosis is likely to be key to the successful initiation of necessary treatment in close-knit communities.

The generalizability of our results may be limited, and replication of results in a more diverse sample is required. Our study population consisted of men who lived in defined communities, were diagnosed late (CD4 count ≤200 cells/mm³), and were eligible for treatment at time of diagnosis, and likely symptomatically in evident need of treatment. Further research is required to assess whether a similar intervention would work on: female PWID; PWID previously diagnosed HIV-infected but not on treatment; PWID whose community is not defined by geography; PWID who do not identify with a community; PWID who may be newly HIV-infected and non-symptomatic; and PWID in other regions of the world. Other limitations of our findings are that our eligibility criteria for this sub-analysis depended on accurate self-report of prior knowledge of one’s HIV-infection.

## Conclusions

With the ever-looming concerns of government cutbacks in funding for HIV prevention and care programs, and the potential of The Presidents Emergency Plan for AIDS Relief (PEPFAR) scale back in Vietnam, this work provides evidence and a framework for effectively improving access and coverage of ART among PWID. The potential for scale-up and sustainability of this multi-level approach should now be investigated with implementation science studies that address barriers to scale-up including need for staff training, lack of leadership commitment and designs that can evaluate implementation outcomes such as uptake and intervention fidelity. In future research it would be important to examine in detail mechanisms of behavior change on the community and individual level and whether these changes are sustained after the intervention. Additionally future investigation into the effect of this multi-level intervention on ART adherence is also warranted. It may also be useful to examine who to target in the community and the necessary percentage of community members to train. These data may also support changing recommendations for referring and enrolling HIV-infected patients into ART early and regardless of symptoms or CD4 count, given the extra time and support that some individuals may need to successfully initiate and continuously engage in HIV care and treatment.

In summary, these findings support those who have highlighted the importance of including behavioral components synergistically at both the individual and community level in combination prevention strategies.[40, 45]

## Supporting Information

S1 Fig The sub-districts of Thai Nguyen, Vietnam, which were enrolled into ACCEPT PROJECT, by trial arm. (JPG)

## Abbreviations

ART: antiretroviral therapy
CIF: cumulative incidence function
CI: confidence interval
PWID: people who inject drugs
OST: opioid substitution therapy
NSP: needle and syringe programs
MMT: Methadone Maintenance Therapy
VCT: voluntary counseling and testing
HTC: HIV testing and counseling
SD: Standard Deviation
