# Supplementary figures and images for: The Effect of a Multi-Level Intervention on the Initiation of Antiretroviral Therapy (ART) among HIV-Infected Men Who Inject Drugs and Were Diagnosed Late in Thai Nguyen, Vietnam
Source: PLoS One. 2016 Aug 31;11(8):e0161718. doi: 10.1371/journal.pone.0161718 (PMC5007027; doi:10.1371/journal.pone.0161718)

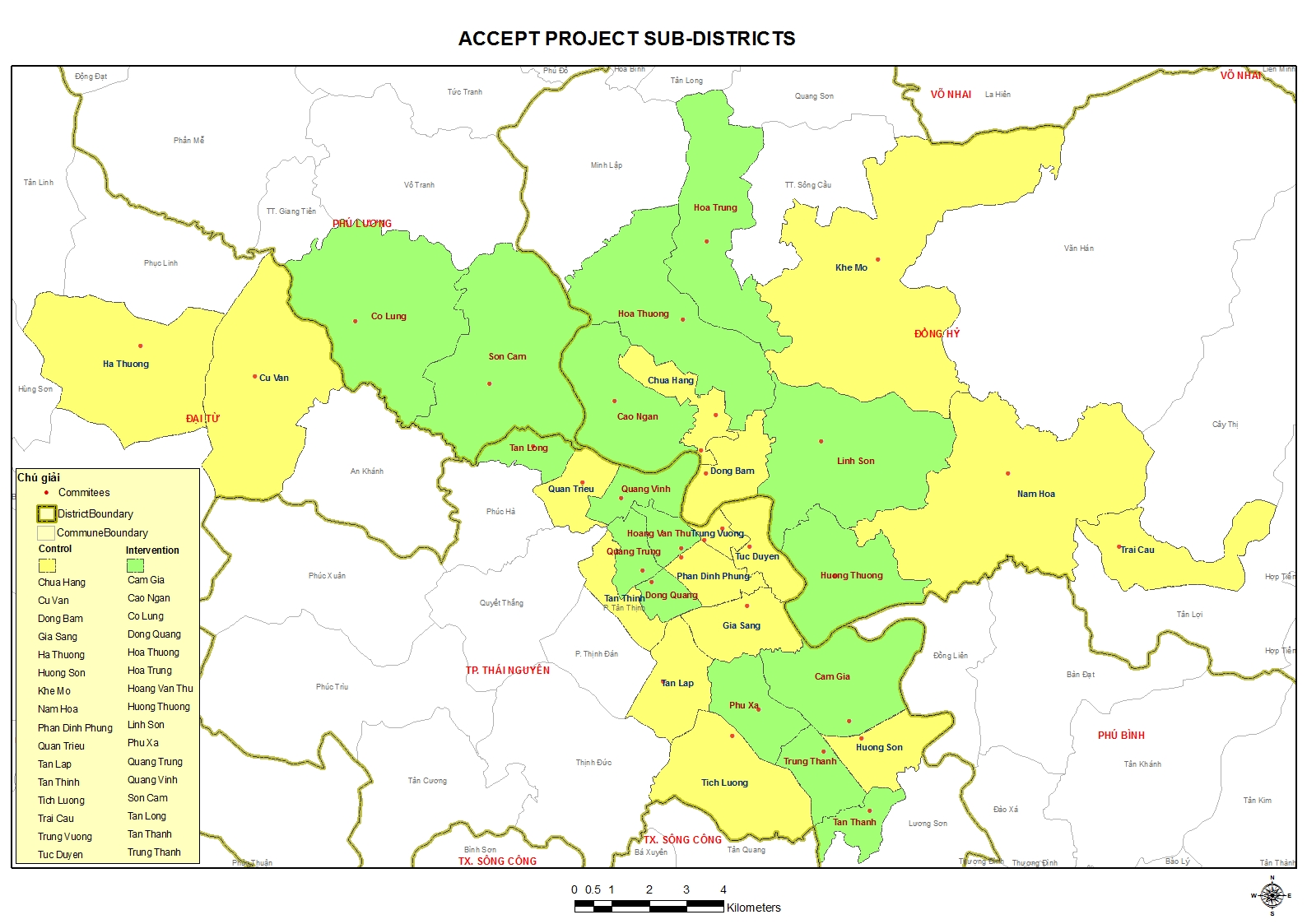

Supplement: S1 Fig — (JPG) [file pone.0161718.s001.jpg]
